# A model-integrated multipoint Bayesian analysis of hypertension in the Framingham Heart Study data finds little evidence of linkage

**DOI:** 10.1186/1471-2156-4-S1-S75

**Published:** 2003-12-31

**Authors:** Mark W Logue, Rhinda J Goedken, Veronica J Vieland

**Affiliations:** 1 Division of Statistical Genetics, Department of Biostatistics, College of Public Health, University of Iowa, Iowa City, Iowa, USA; 2 Center for Statistical Genetics Research, College of Public Health and Roy J. & Lucille A. Carver College of Medicine, University of Iowa, Iowa City, Iowa, USA; 3 Department of Psychiatry, Roy J. & Lucille A. Carver College of Medicine, University of Iowa, Iowa City, Iowa, USA

## Abstract

This Genetic Analysis Workshop 13 contribution presents a linkage analysis of hypertension in the Framingham data based on the posterior probability of linkage, or PPL. We dichotomized the phenotype, coding individuals who had been treated for hypertension at any time, as well as those with repeated high blood pressure measurements, as affected. Here we use a new variation on the multipoint PPL that incorporates integration over the genetic model. PPLs were computed for chromosomes 1 through 5, 11, 14, and 17 and remained below the 2% assumed prior probability of linkage for 73% of the locations examined. The maximum PPL of 4.5% was obtained on chromosome 1 at 178 cM. Although this is more than twice the assumed prior probability of linkage, it is well below a level at which we would recommend committing substantial additional resources to molecular follow-up. While the PPL analysis of this data remains inconclusive, Bayesian methodology gives us a clear mechanism for using the information gained here in further studies.

## Background

The posterior probability of linkage, or PPL, has several advantages over other parametric methods [1]. First, it is directly interpretable as the probability of linkage to a specific marker or location along the genome. Second, as a Bayesian statistic, can incorporate any prior information that the researcher might have before the outset of a study. Third, it provides an easy way to combine the information from several studies without pooling the data.

Here we exploit a fourth feature of the PPL, which allows us to integrate over the parameters of the trait model, resulting in a statistic that is model-free in the sense that it does not fix trait parameters at specific values. Thus, we are able to allow for heterogeneity, reduced penetrance, and phenocopies. Additionally, using integration in this way allows us to avoid the inflationary effects on the likelihood of maximizing over multiple parameters. This makes the model-integrated PPL an ideal tool for the analysis of complex diseases such as hypertension.

This also represents the first analysis using a model-integrated version of the PPL based on multipoint likelihoods. The multipoint PPL gives us an indication of whether or not there is a disease gene close to each position on the chromosome [2], in contrast to the two-point version [1,3-5], which estimates the probability of linkage to each marker individually.

## Methods

### Data description

When analyzing the Framingham Heart Study data set, two issues needed attention. First, it was necessary to establish a dichotomous phenotype definition that reliably captured the information from the multiple measurements. Our phenotype definition combined the "treated for hypertension" variable and the "high blood pressure" variable. An individual treated for hypertension at any time point was called affected. Of those not treated for hypertension, individuals having high blood pressure at four or more time points were coded as affected; the majority (57%) of people who had four or more high blood pressure readings were also treated for hypertension at some point. Of the people who had three or fewer high blood pressure readings, those who had fewer than eight recorded measurements were coded as unknown. Those with eight or more measurements were coded as unaffected. Individuals with no history of treatment and no blood pressure measurements were coded as unknown. This definition was intended to minimize the number of misclassified individuals by coding those without a clear propensity for high or normal blood pressure as unknown.

The large amounts of relatively sparse data pose a second problem. First, 144 pedigrees with fewer than three affected members (according to our phenotype definition) were removed. This could have the effect of eliminating sporadic cases, resulting in a more homogeneous data set, but primarily this was done to speed the calculations. Of the remaining 190 pedigrees, GENEHUNTER [see below] was unable to use 60 due to their structure. Therefore, we split these pedigrees into their constituent nuclear families using MEGA2 (Version 2.3, [6]). (See [7] for potential pitfalls.) The final data set was made up of 620 pedigrees, with an average pedigree size of 5.96. Of the 3695 individuals in the data set, 1312 were coded as affected, 232 were coded as unaffected, and the remaining 2151 were coded as unknown.

Due to the computational complexity of the methods, eight chromosomes were chosen for analysis. PPLs are presented for chromosomes 1 through 5, 11, 14, and 17.

### Statistical methods

The statistical methods used in this paper are a logical extension of previous work on the PPL. Initially, the PPL was developed as a two-point statistic based on a heterogeneity LOD score [1,4-6]. In the Genetic Analysis Workshop 12 this was expanded to include a multipoint version [2]. Recently the two-point PPL has been adapted to allow integration over the parameters of the genetic model [8], and used in a genome screen for specific language impairment [9]. In this paper we have combined these two variants to produce a multipoint version of the model-integrated PPL. The model integrated multipoint PPL at position t_0 _can be written:



where *t *is the cM position; P_L _is the assumed prior probability of linkage; *k *is the size of the moving window (see below); α is the admixture parameter [10]; and *g *is a vector of parameters that describe the genetic model (disease allele frequency and three penetrances). The HLOD is the multipoint heterogeneity (admixture) LOD score [11] as a function of *t*, α and *g*. The prior distributions for *g *and α are represented by *h*(*g*) and *h*(α).

The moving-window prior *h*(|*t *- *t*_0_|) on the disease gene location is used to summarize the probability that a risk locus is close to each position. This prior is constructed to mirror the one used in the two-point case, and places positive probability at all points within 44 cM, with 95% of the probability concentrated within ± 5 cM [2]. Multipoint LOD scores are computed by a series of programs that make repeated calls to GENEHUNTER [12], varying the values of the genetic model in each run. HLODs are computed every cM at 20 different α values for 1650 different genetic models (see [9] for a complete description of the model grid). The average likelihood ratio (10^*HLOD*(*t*, α, *g*)^) is computed at each position to approximate the integration of the likelihood surface over the genetic model. A 2% prior probability was assumed [13].

## Results

**Figure 1:**
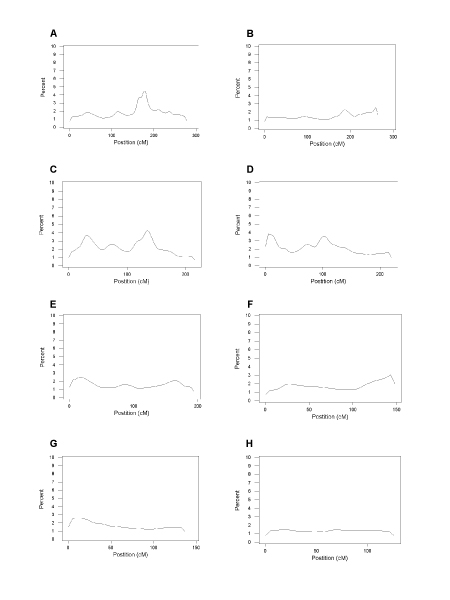
Chromosome PPLs. A, chromosome 1; B, chromosome 2;C, chromosome 3; D, chromosome 4; E, chromosome 5; F, chromosome 11;G, chromosome 14; H, chromosome 17

73% of the PPLs were below the prior probability of linkage. The maximum PPL of 4.5% was observed on chromosome 1 at 178 cM. The next highest PPL of 4.2% was achieved on chromosome 3 at 135 cM. The PPL was below 4% on the remaining chromosomes. The graphs of the PPLs for chromosomes 1 through 5, 11, 14, and 17 are presented in Figure 1.

## Conclusions

At this point we have no compelling evidence for a hypertension-predisposing gene on any of the chromosomes we examined. Our largest PPLs of 4.5% and 4.2% were observed on chromosomes 1 and 3. A PPL of 4% indicates a location that is twice as likely to be linked to the trait being studied than one chosen at random. However, this is well below the level at which we would suggest committing substantial additional resources to fine mapping or molecular work.

Overall, PPLs obtained do not differ much from the assumed prior probability of linkage. This indicates that we have little evidence for or against linkage across the genome. The PPL has the property that as the amount of genetic information increases the PPL converges to 1 under linkage and 0 under no linkage [4]. One explanation for this is a lack of sufficient data. Further work with this data set could involve performing the analysis using a program other than GENEHUNTER, which was chosen for computational convenience. It has been shown that the pedigree trimming performed by GENEHUNTER and the splitting of pedigrees into nuclear families can cause low power and misleading results [7]. Furthermore, as additional data become available, the Bayesian nature of the PPL yields a natural way to update the information from one study to the next. The posterior distributions computed in this analysis could be used as the prior information for further studies of hypertension. Alternatively, our definition of the phenotype may not be genetically relevant.
